# Role of Caffeine Intake on Erectile Dysfunction in US Men: Results from NHANES 2001-2004

**DOI:** 10.1371/journal.pone.0123547

**Published:** 2015-04-28

**Authors:** David S. Lopez, Run Wang, Konstantinos K. Tsilidis, Huirong Zhu, Carrie R. Daniel, Arup Sinha, Steven Canfield

**Affiliations:** 1 Division of Epidemiology, Human Genetics and Environmental Sciences, University of Texas- Houston School of Public Health, Houston, TX, United States of America; 2 Division of Urology, University of Texas- Houston Medical School, Houston, TX, United States of America; 3 Department of Hygiene and Epidemiology, University of Ioannina School of Medicine, Ioannina, Greece; 4 Department of Epidemiology, University of Texas- MD Anderson Cancer Center, Houston, TX, United States of America; 5 Division of Biostatistics, University of Texas- Houston School of Public Health, Houston, TX, United States of America; Leibniz Institute for Neurobiology, GERMANY

## Abstract

**Objectives:**

Caffeine is consumed by more than 85% of adults and little is known about its role on erectile dysfunction (ED) in population-based studies. We investigated the association of caffeine intake and caffeinated beverages with ED, and whether these associations vary among comorbidities for ED.

**Material and Method:**

Data were analyzed for 3724 men (≥20 years old) who participated in the National Health and Nutrition Examination Survey (NHANES). ED was assessed by a single question during a self-paced, computer-assisted self-interview. We analyzed 24-h dietary recall data to estimate caffeine intake (mg/day). Multivariable logistic regression analyses using appropriate sampling weights were conducted.

**Results:**

We found that men in the 3^rd^ (85-170 mg/day) and 4^th^ (171-303 mg/day) quintiles of caffeine intake were less likely to report ED compared to men in the lowest 1^st^ quintile (0-7 mg/day) [OR: 0.58; 95% CI, 0.37–0.89; and OR: 0.61; 95% CI, 0.38–0.97, respectively], but no evidence for a trend. Similarly, among overweight/obese and hypertensive men, there was an inverse association between higher quintiles of caffeine intake and ED compared to men in the lowest 1^st^ quintile, *P≤0*.*05* for each quintile. However, only among men without diabetes we found a similar inverse association (*P_trend_* = 0.01).

**Conclusion:**

Caffeine intake reduced the odds of prevalent ED, especially an intake equivalent to approximately 2-3 daily cups of coffee (170-375 mg/day). This reduction was also observed among overweight/obese and hypertensive, but not among diabetic men. Yet, these associations are warranted to be investigated in prospective studies.

## Introduction

In the US, the prevalence of erectile dysfunction (ED) in men aged ≥20 years is 18.4% suggesting that more than 18 million are affected [1, 2]. Among older men, these numbers significantly increase affecting their overall quality of life [3]: at age 40, approximately 44% are affected and this number increases nearly to 70% by age of 70 [2, 4]. The economic burden of ED is unclear, yet studies have shown that the cost of treatment could reach $15 billion if all men seek treatment [5, 6].

Cardiovascular risk factors like physical inactivity, alcohol consumption and smoking have been suggested to increase the risk of ED [7, 8]. Yet, little is known about other factors that could have a potential benefit on ED such as caffeine intake [9–12]. It was previously hypothesized that coffee and/or caffeine initiates a series of pharmacological effects that lead to the relaxation of the cavernous smooth muscle and that subsequently could improve ED [13]. Caffeine was recently reported to be consumed by more than 85% of US adults, and it is obtained mainly from dietary fluid sources, such as coffee, tea, soda, and energy/sport drinks. About two thirds of American adults drink coffee on a daily basis, 52% of the US male population drinks at least one glass of soda per day, while over 50% drinks tea on any given day, and 17% consumes energy and sport drinks more than three times per week [14]. Coffee, and its most studied component, caffeine, have been implicated in potential health benefits due to the rich sources of antioxidants and anti-inflammatory compounds contained in this beverage [14, 15].

The prevalence of overweight/obesity, hypertension and diabetes is increasing dramatically among US men, and previous studies have linked them with ED [16–20]. Yet, there is a paucity of research on the interplay between these comorbidities, caffeine intake and ED. Therefore, the aims of this study are to investigate the association of caffeine intake and caffeinated beverages with ED, and to compare whether these associations vary among overweight/obese, hypertensive and diabetic men in a nationally representative sample of the US adult population.

## Methods

### Study Population

The National Health and Nutrition Examination Survey (NHANES) is a program of studies undertaken by the National Center for Health Statistics (NCHS) of the United States (US) Centers for Disease Control and Prevention (CDC) to assess the health and nutritional status of adults and children in the US. Continuous NHANES used a multistage, stratified and clustered probability sampling in which Mexican-Americans, non-Hispanic blacks, and the elderly were oversampled to ensure adequate samples sizes and to represent the total US civilian, non-institutionalized population [21]. For the purpose of this study, we combined data from continuous NHANES waves 2001–2002 and 2003–2004 because information on ED was reported only on those years.

### Assessment of Erectile Dysfunction

NHANES participants were in a private room using self-paced audio computer-assisted self-interview system that enabled them to both hear questions through earphones and read questions on the computer related to ED. To assess erectile dysfunction, men (≥20 years) were asked the following question that has been previously validated and suggested to be added in large ongoing national epidemiologic surveys to provide needed information related to the prevalence of ED [22], *“Many men experience problems with sexual intercourse*. *How would you describe your ability to get and keep an erection adequate for satisfactory intercourse*? The following answers were provided, *“would you say that you are*… *always or almost always able*, *usually able*, *sometimes able*, *or never able*? For the purpose of this study, we defined and dichotomized positive ED from the answers “sometime able” or “never able” to keep an erection, and subsequently negative ED was derived from the answers “almost always able” or “usually able” to maintain an erection [2]. In this study we excluded men with the following conditions as they could influence the condition of ED: men who were diagnosed with prostate cancer (n = 94), or underwent surgery/radiation treatment (n = 85) for the same disease. We also excluded men with implausible daily calorie intakes (below 800 kcal or above 5,000 kcal) (n = 285) leaving a total sample size of 3,724 men with valid data on ED.

### Assessment of caffeine and dietary data

The U.S. Department of Agriculture developed and validated a multiple-pass dietary recall method for NHANES to collect dietary data [23]. Participants reported all food and beverages consumed in two, 24-h dietary recall periods (midnight to midnight). The first one was conducted by dietary research interviewers face-to-face, and the second one was done 3 to 10 days later by telephone. Because NHANES 2001–2002 only included one recall, our analysis was limited to the first-day dietary recall for both NHANES waves 2001–2002 and 2003–2004, which is considered a population-based estimate of daily caffeine intake. After the dietary interviews, USDA’s Food and Nutrient Database for Dietary Studies 5.0 (2012) was used to code dietary intake data and calculate nutrient intakes [23]. Based on the quantity of food and beverages reported and the corresponding nutrient contents by the National Center for Health Statistics (NCHS), the caloric content and other nutrients derived from each consumed food and beverage item were calculated [23, 24]. Data on caffeine intake (mg/day), plain and tap water (gm), and alcohol (gm) was obtained from the Total Nutrient File, which contains summed nutrients for an individual from all food and beverages provided on the dietary recall [25]. Information on specific caffeinated beverages was obtained from the Individual Foods files [23]. We identified four beverages, coffee, total soda (regular and low-calorie), tea and energy and sport drinks, which were dichotomized (“Yes” / “No”) for their intake on any given day. We examined caffeine intake in quintiles (mg/day), lowest quintile (0–7), 2^nd^ quintile (8–84), 3^rd^ quintile (85–170), 4^th^ quintile (171–303), and highest quintile (304–700). Total water intake categorized and defined by combining plain and tap water and it was included in multivariable models, while alcohol consumption was kept continuous.

### Assessment of covariates

Age, race/ethnicity, smoking status, education and physical activity during the past 30 days (moderate and vigorous) were self-reported during the NHANES interview. NHANES categorizes race/ethnicity as non-Hispanic white, non-Hispanic black, Mexican American, and other (other Hispanics and all others). Participants were classified as never, former and current smokers from self-reported information; participants were asked if they had smoked more than 100 cigarettes in their lifetime and if they were current smokers; serum cotinine was measured using high performance liquid chromatography/atmospheric-pressure ionization tandem mass spectrometry [26]. Current smokers consisted of those who self-reported smoking habits, smoked more than 100 cigarettes in their lifetime, and cotinine was ≥ 10 ng/mL (actively exposed). Vigorous physical activity was obtained from the questions on whether participants did any activity that caused heavy sweating or large increases in breathing or heart rate (e.g., swimming, aerobics, or fast cycling), while moderate physical activity was determined from the questions on whether they did any activities that caused light sweating or a moderate increase in the heart rate, such as playing golf, dancing, bicycling for pleasure, or walking. Three readings of systolic and diastolic blood pressure were obtained from participants who attended the mobile examination center. We used the average of those three measurements (≥140/90 mmHg). We also considered the current use of antihypertensive medication treatment or being “told by a doctor you have hypertension” as an indication of high blood pressure (hypertension). Body mass index (BMI) was calculated from measured weight and height (weight in kilograms divided by height in meters squared). Type 2 diabetes status was defined from ≥126 mg/dl of fasting plasma glucose, medication treatment or being “told by a doctor you have diabetes or sugar diabetes.” Fasting plasma glucose concentration was measured in the morning session after an overnight fast of at least 8 h [27], details related to the laboratory procedures of that is found elsewhere [21].

### Statistical analysis

Sampling weights were applied to take into account selection probabilities, over-sampling, non-response, and differences between the sample and the total US population. Adjusted odds ratios and 95% confidence intervals (CI) for ED using weighted logistic regression models were estimated in relation to quintiles of caffeine intake, binary variables of caffeinated beverages and its combinations. We did not attempt to classify “coffee *plus* tea” (or any of the beverage combinations) intake according to caffeine intake (mg/day) content due to missing information about specific beverage consumption, missing information on caffeine (mg/day), or discrepancy between caffeine (mg/day) values and reporting of specific beverage consumption. In “Model 1,” we only adjusted for age, while in “Model 2” we included the variables vigorous and moderate physical activity, age (continuous), smoking status, education, race/ethnicity, obesity (BMI ≥ 30 kg/m^2^), total water intake (plain and tap; continuous), total energy (continuous) and alcohol (continuous).

Stratified analyses were conducted by binary overweight/obesity (BMI ≥25 kg/m^2^), type 2 diabetes and hypertension because these variables are known to modify ED. Multivariable models included the variables vigorous and moderate physical activity, age, smoking status, education, race/ethnicity, intake of total water, total energy, alcohol and obesity (removed when stratified by overweight/obesity). Statistical interaction tests were carried out by using the ordinal variables for caffeine intake, binary variables for caffeinated beverages, binary variables for the potentially modifying factors and their product terms. The statistical significance of the interaction terms was evaluated by the Wald test. All p-values were two-sided; alpha = 0.05 was considered the cut-off for statistical significance. All statistical analyses were performed using STATA version 12.0 (College Station, TX).

## Results

The distribution of baseline characteristics in the study population after applying samples weights is shown in Table 1. The mean age was 49 years, and the majority of the participants were white (54%). The prevalence of overweight and obesity based on BMI ≥25–29.99 and ≥30 kg/m^2^, respectively, was 40.9% and 30.7%. Fifty-one percent of the participants were hypertensive and 12.4% were diabetic. Twenty-six percent of the participants had some college and 24.7% had a high school diploma or GED equivalent, 34.6% were current smokers, 49.9% performed moderate physical activity, while a 64.7% reported a vigorous physical activity. Mean value for serum cotinine was 70.0 ng/mL, 1900.7 gm for total water intake, 2434.8 kcal for energy, 15.4 gm for alcohol and 188.3 mg/d for total caffeine intake. Caffeinated beverages showed 55.4% for coffee, 20.6% for tea, 58.5% for total soda, and 3.1% for energy and sport drinks.

**Table 1:** Selected characteristics of the US population of adult men 20 years or older, NHANES 2001–2004.

Characteristics	Unweighted sample size	Mean or percentage (SE)^†^
Age, years	3724	49.4 (18.4)
Race/ethnicity, %		
Mexican-American	774	20.8
Non-Hispanic White	2016	54.1
Non-Hispanic Black	689	18.5
Other	245	6.6
Education, %		
Less than 9^th^ grade	489	13.1
9^th-^ 11^th^ grade	539	14.5
High school grad / GED or equivalent	920	24.7
Some College or associate degree	979	26.3
College Graduate or above	797	21.4
Cigarette smoking, %		
Never	1371	36.8
Former	1063	28.6
Current^‡^	1287	34.6
Serum cotinine, ng/mL	3579	70.9 (137.3)
Body mass index, kg/m^2^, %		
<25	1040	28.4
≥25–29.99	1498	40.9
≥30	1124	30.7
Type 2 Diabetes, %		
No	3263	87.6
Yes	461	12.4
Hypertension, %		
No	1809	48.6
Yes	1911	51.4
Physical Activity Status, %		
Moderate		
No	1818	50.1
Yes	1816	49.9
Vigorous		
No	1261	35.3
Yes	2316	64.7
Total water intake, gm/day	2113	1900.7 (2192.5)
Total energy, kcal/day	3724	2434.8 (910.1)
Alcohol, gm/day	3724	15.4 (35.6)
Total caffeine intake, mg/day	3724	188.3 (246.6)
Coffee consumption, %		
No	1663	44.6
Yes	2061	55.4
Tea consumption, %		
No	2956	79.4
Yes	768	20.6
Total soda consumption, %		
No	1544	41.5
Yes	2180	58.5
Energy and sport drinks, %		
No	3608	96.9
Yes	116	3.1
Coffee *plus* tea, %		
No	1315	35.3
Yes	2409	64.7
Coffee *plus* tea and soda, %		
No	404	10.9
Yes	3320	89.1
Coffee *plus* tea, soda, and energy and sport drinks, %		
No	381	10.2
Yes	3343	89.7

SE: standard error

^†^Sampling weights were applied

^‡^Self-reported smoking habits, smoked more than 100 cigarettes in their lifetime, and cotinine was ≥ 10 ng/mL (actively exposed).

**Fig 1:**
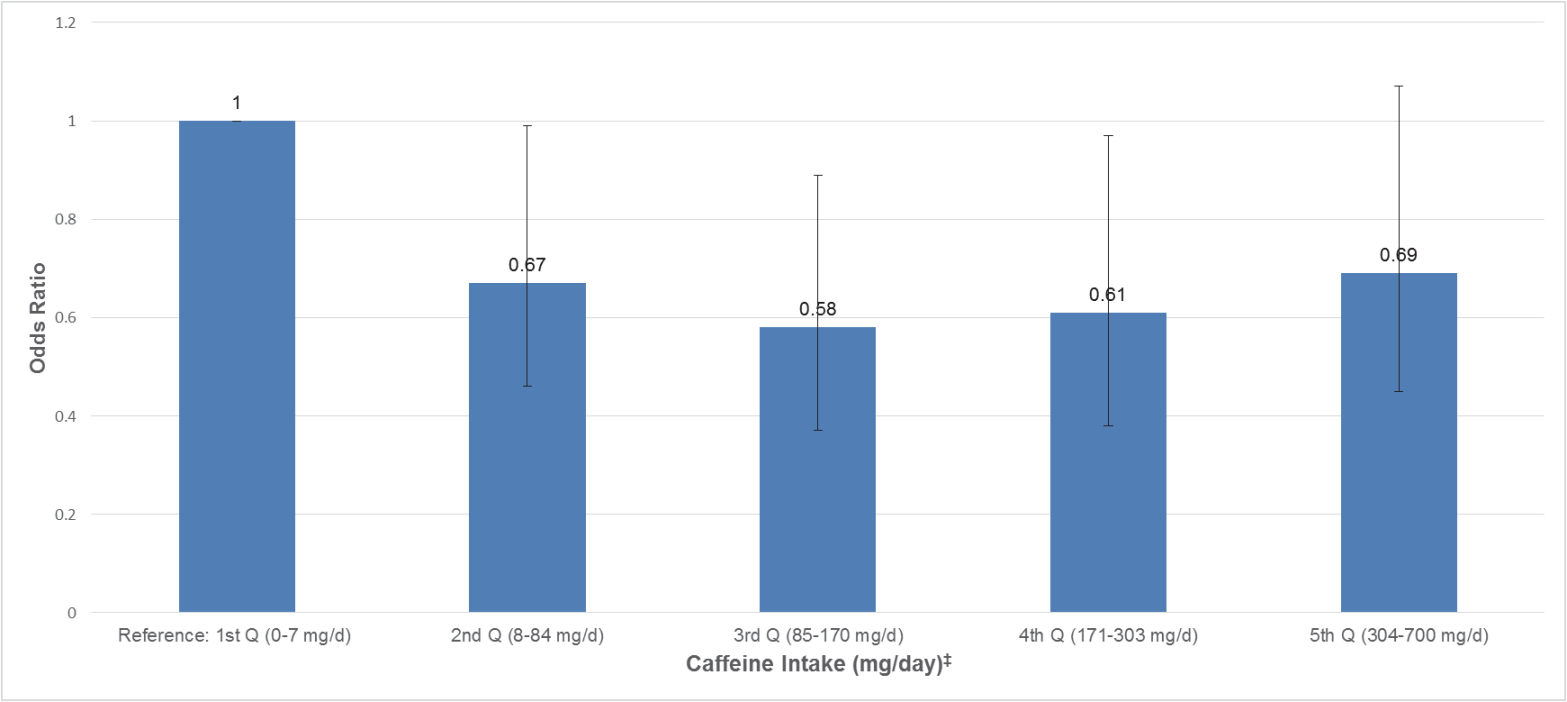
Association of caffeine intake with erectile dysfunction in NHANES 2001–2004 (n = 3724). ^†^Adjusted for age, vigorous and moderate physical activity, smoking status, education, race/ethnicity, obesity (BMI ≥ 30 kg/m^2^), total water intake (plain and tap), total energy (continuous), alcohol (continuous). Error bars represent 95% confidence intervals. *P*_*trend*_ *= 0*.*19*. ^*^Erectile dysfunction was defined as “sometimes” or “never” able to maintain an erection for satisfactory sexual intercourse. ^‡^Approximately 170–375 mg/day of caffeine intake is equivalent to 2–3 cups of coffee.

In Fig 1, after adjusting for vigorous and moderate physical activity, age, smoking status, education, race/ethnicity, obesity, total water intake, total energy, and alcohol, we found that men in the 3^rd^ quintile (85-170mg/day) and 4^th^ quintile (171–303 mg/day) of total caffeine intake were less likely to report ED compared to men in the 1^st^ quintile (0–7 mg/day) [OR: 0.58; 95% CI, 0.37–0.89; and OR: 0.61; 95% CI, 0.38–0.97, respectively]. Yet, we did not find a trend in this association (*P*_*trend*_ = 0.19).

**Fig 2:**
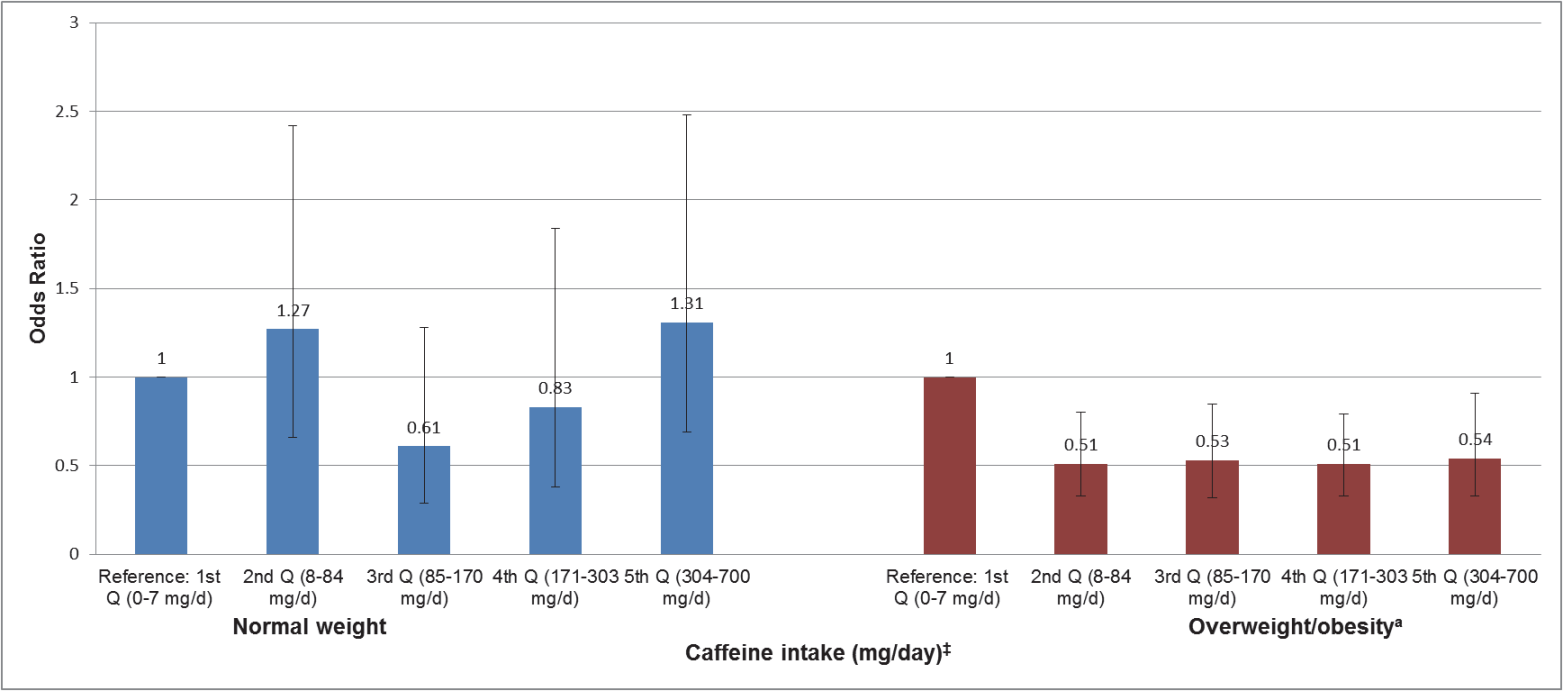
Association of caffeine intake with erectile dysfunction among normal weight and overweight/obese men in NHANES 2001–2004. ^†^Adjusted for age, vigorous and moderate physical activity, smoking status, education, race/ethnicity, obesity (BMI ≥ 30 kg/m^2^), total water intake (plain and tap), total energy (continuous), alcohol (continuous). Error bars represent 95% confidence intervals. *P*_*trend*_ *= 0*.*08 and P*_*interaction*_ *= 0*.*09*. ^*^Erectile dysfunction was defined as “sometimes” or “never” able to maintain an erection for satisfactory sexual intercourse. ^‡^ Approximately 170–375 mg/day of caffeine intake is equivalent to 2–3 cups of coffee. ^a^Overweight/obesity = body mass index ≥ 25 kg/m^2^.

**Fig 3:**
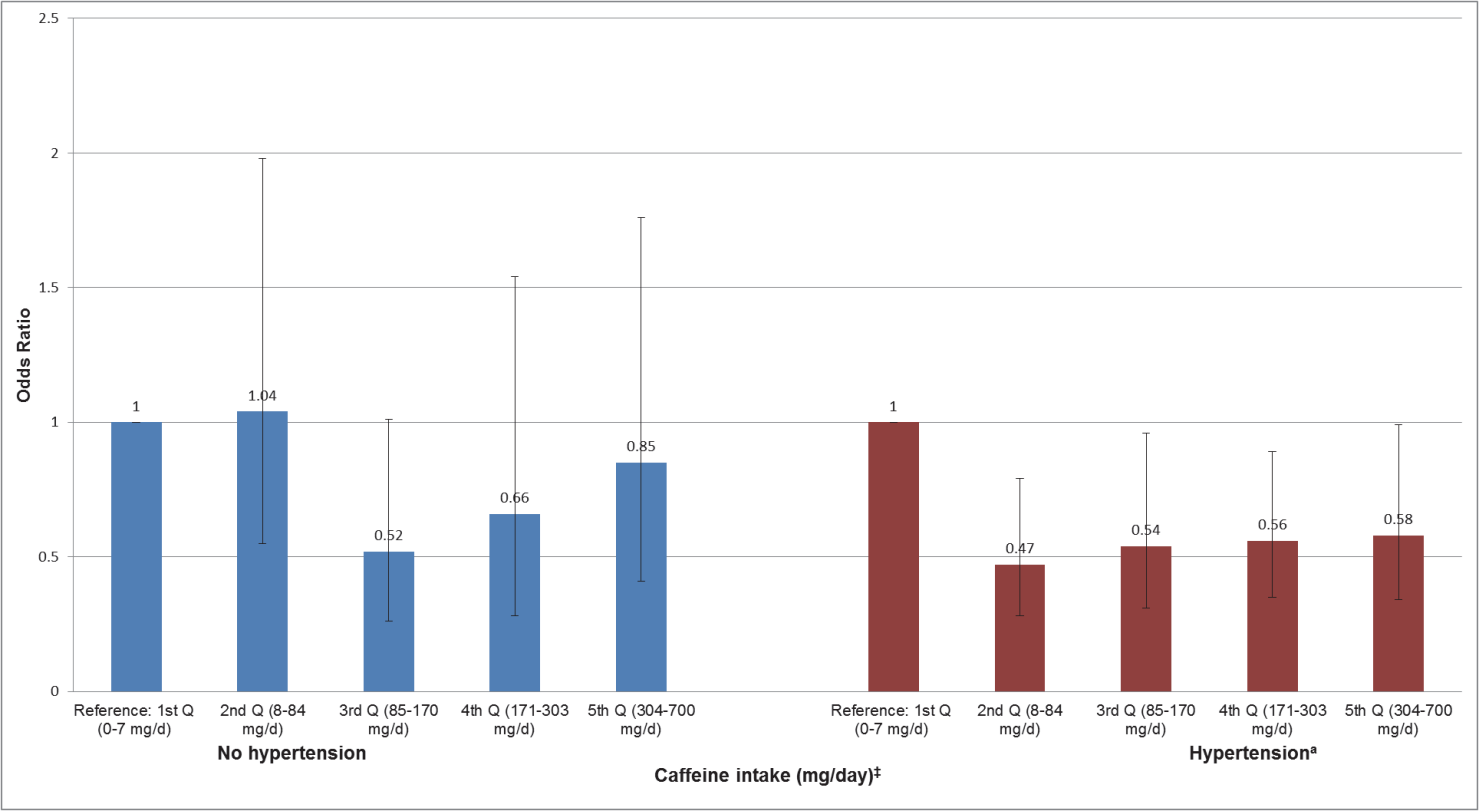
Association of caffeine intake with erectile dysfunction among men with and without hypertension in NHANES 2001–2004. ^†^Adjusted for age, vigorous and moderate physical activity, smoking status, education, race/ethnicity, obesity (BMI ≥ 30 kg/m^2^), total water intake (plain and tap), total energy (continuous), alcohol (continuous). Error bars represent 95% confidence intervals. *P*_*trend*_ *= 0*.*13 and P*_*interaction*_ *= 0*.*30*. ^*^Erectile dysfunction was defined as “sometimes” or “never” able to maintain an erection for satisfactory sexual intercourse. ^‡^ Approximately 170–375 mg/day of caffeine intake is equivalent to 2–3 cups of coffee. ^a^Hypertension = systolic/diastolic blood pressure ≥140/90 mmHg, self-reported diagnosis, or medication treatment.

**Fig 4:**
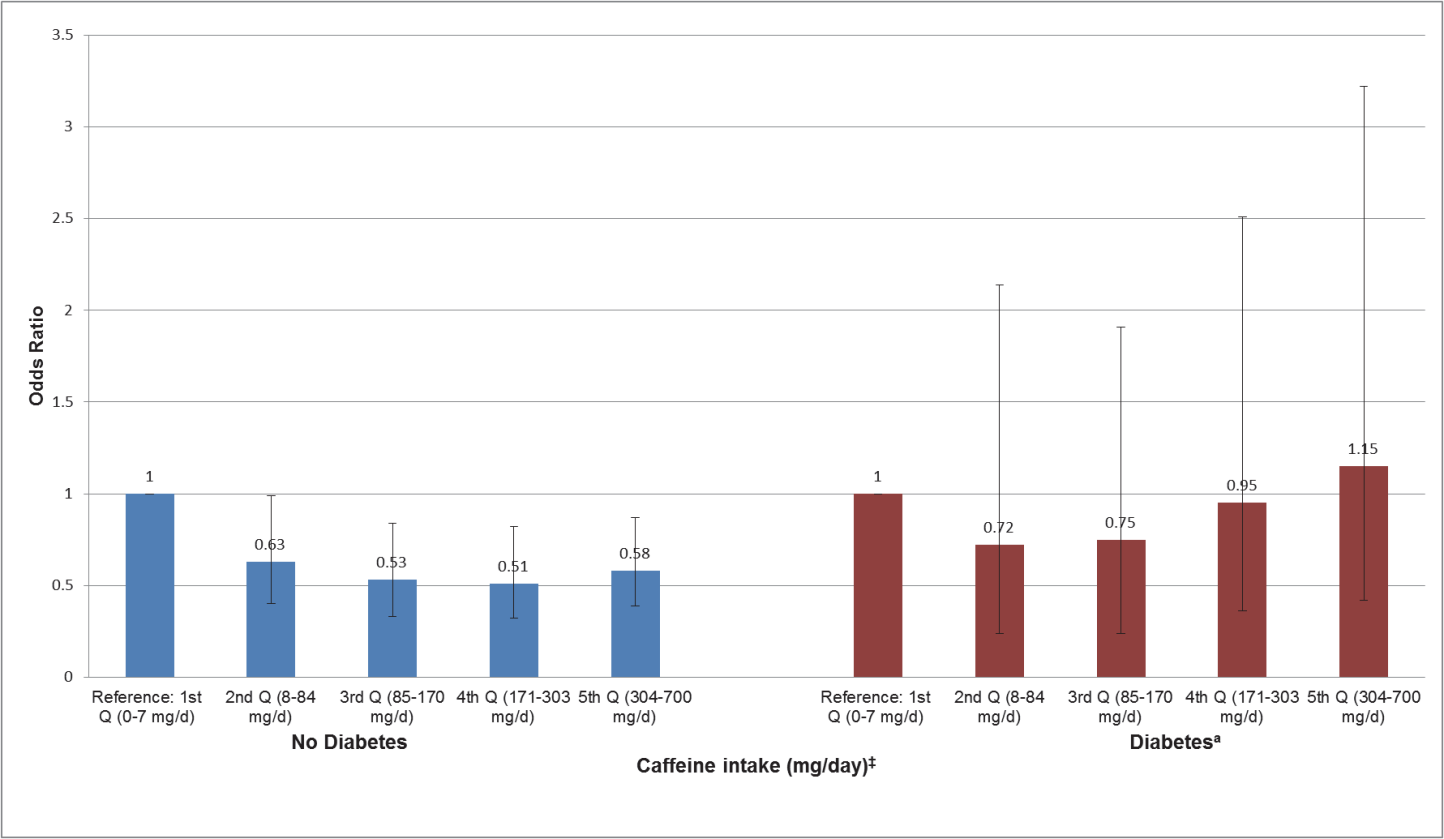
Association of caffeine intake with erectile dysfunction among men with and without diabetes in NHANES 2001–2004. ^†^Adjusted for age, vigorous and moderate physical activity, smoking status, education, race/ethnicity, obesity (BMI ≥ 30 kg/m^2^), total water intake (plain and tap), total energy (continuous), alcohol (continuous). Error bars represent 95% confidence intervals. *P*_*trend*_ *= 0*.*57 and P*_*interaction*_ *= 0*.*65*. ^*^Erectile dysfunction was defined as “sometimes” or “never” able to maintain an erection for satisfactory sexual intercourse. ^‡^ Approximately 170–375 mg/day of caffeine intake is equivalent to 2–3 cups of coffee. ^a^Diabetes = fasting plasma glucose ≥126 mg/dl, self-reported diagnosis, or medication treatment.

In stratified analyses, we investigated the associations of total caffeine intake with ED among overweight/obese (BMI ≥ 25 kg/m^2^), hypertensive and diabetic men (Figs 2–4). We did not find any significant association among men with normal weight (BMI < 25 kg/m^2^), normal blood pressure, and diabetes. However, among overweight/obese and hypertensive men, we found that men in the 2^nd^ (8–84 mg/day), 3^rd^ (85–170 mg/day), 4^th^ (171–303 mg/day) and 5^th^ (304–700 mg/day) quintiles of total caffeine intake had a reduced prevalence of ED than men in the lowest quintile (0–7 mg/day) (*P* ≤ 0.05 for each quintile) (Figs 2 and 3). Yet, there was no evidence for a statistically significant trend (*P*_*trend*_ ≥ 0.05) and interaction effect (*P*_*interaction*_ ≥ 0.05) for any of the associations. Among men without diabetes (Fig 4), we found that men in every caffeine intake quintile reported a lower likelihood of prevalent ED showing a significant trend for this association (*P*_*trend*_ = 0.01). Yet, the interaction effect did not reach statistical significance (*P*_*interaction*_ = 0.65). The association of caffeine intake with ED was also analyzed in age categories (20–39, 40–49, 50–59, and ≥60 years old), however, no statistical significant associations were found (data not shown).

We also analyzed the association of caffeinated beverages with ED and whether these associations varied by comorbidities for ED. The results are presented in an Online Supplement (S1–S4 Tables). In general, we found few significant, but inconclusive, associations between caffeinated beverages, and its combinations, with ED (S1–S4 Tables).

## Discussion

In the present study, our nationally representative sample of men showed that total caffeine intake was associated with a reduced likelihood to report ED in multivariable analyses. This reduced prevalence of ED was mainly observed when the amount of caffeine intake was between 170 and 375 mg/day, which is approximately equivalent to 2–3 cups of coffee per day. In addition, total caffeine intake seemed to reduce the odds of ED among men who were overweight/obese, hypertensive and non-diabetic. A few caffeinated beverages were inversely associated with ED, yet they were not consistent when stratified by comorbidities for ED.

Our results are consistent with two population-based studies that showed an inverse association between caffeine intake and ED [9, 10]. The suggested biological mechanism [20] is that caffeine triggers a series of pharmacological effects that lead to the relaxation of the penile helicine arteries, and the cavernous smooth muscle that lines cavernosal spaces, thus increasing penile blood flow [13].

However, our findings differ from two previous studies that showed no association between caffeinated drinks (cross-sectional), coffee consumption (prospective) and ED [11, 12]. Compared to these null studies, our study had a larger sample size for the endpoint of interest. In addition, our exposures (total caffeine intake and caffeinated beverages) were derived from a 24-hour dietary recall period, which method has been developed and validated by the US Department of Agriculture to report population-based estimates of daily caffeine intake. Plus, our multiple linear regression models included more confounders, total water intake and total energy, that could possibly mask an association between caffeine intake and ED.

We did not find a significant inverse association between caffeine intake and ED among diabetic men. In an animal study, we previously showed that caffeine consumption improved the erectile function of diabetic rats by up-regulating cavernous cGMP [20]. Diabetes is one of the strongest risk factors for ED and it remains one of the most difficult medical conditions to treat [28]. Thus, it is possible this is one of the reasons we couldn’t find lower prevalence of ED among men with higher intake of caffeine.

Interestingly, our findings showed a lower prevalence of ED among men with caffeine intake, especially when this is equivalent to 2–3 daily cups of coffee (170–375 mg/day). Previously, a similar caffeine intake amount was associated with beneficial effects on cardiometabolic factors and cardiovascular health [14]. In addition, a recent study reported that American men age 35 to 54 years had a caffeine intake of 336 mg per day, which is close to the amount of caffeine we found having a significantly inverse association with ED [29]. Our findings with caffeinated beverages, or its combination, did not follow a consistent pattern as we observed with total caffeine intake (mg/day). Yet, it is possible that due to its collection (consumption on any given day) and dichotomization (“Yes” / “No”) contributed to the inconsistent results we found.

Strengths of this study is the large sample size with a representative sample of the US men population, and the validated dietary recall methodology from NHANES that allows for measurement of caffeine intake from drinks [23, 24] and report population-based estimates of daily caffeine intake. Yet, our study also has limitations; ED is a multifactorial disease and some of its risk factors were not addressed in this study such as cardiovascular diseases; NHANES is a cross-sectional study, therefore, the association we found between caffeine intake and ED impede us to infer causality or much less suggest any clinical practice change. Plus, there is an inherent bias in the use of surveys for data collection. Thus, these findings should be confirmed in prospective studies.

## Conclusion

In general we found a reduced likelihood to report ED among men with caffeine intake, especially with 2–3 daily cups of coffee, which is approximately 170–375 mg/day. Interestingly, we found differences in the inverse associations of caffeine intake and caffeinated beverages among overweight/obese, non-diabetic and hypertensive men.

## Supporting Information

S1 Table Association of caffeine intake and caffeinated beverages with erectile dysfunction^β^ in NHANES 2001–2004 (n = 3724). ^β^Erectile dysfunction was defined as “sometimes” or “never” able to maintain an erection for satisfactory sexual intercourse. ^†^Model 1- Adjusted for age only. ^‡^Model 2- Adjusted for age, vigorous and moderate physical activity, smoking status, education, race/ethnicity, obesity (BMI ≥ 30 kg/m^2^), total water intake (plain and tap), total energy (continuous), alcohol (continuous). ^£^ Approximately 170–375 mg/day of caffeine intake is equivalent to 2–3 cups of coffee. ^a^ *P* ≤ 0.05 ^b^ *P ≤* 0.01
(DOC)

S2 Table Association of caffeine intake and caffeinated beverages with erectile dysfunction^β^ among normal weight and overweight/obese men in NHANES 2001–2004. ^β^Erectile dysfunction was defined as “sometimes” or “never” able to maintain an erection for satisfactory sexual intercourse. ^‡^Adjusted for age, vigorous and moderate physical activity, smoking status, education, race/ethnicity, total water intake (plain and tap), total energy (continuous), alcohol (continuous). ^£^Approximately 170–375 mg/day of caffeine intake is equivalent to 2–3 cups of coffee. ^a^ *P* ≤ 0.05 ^b^ *P ≤* 0.01
(DOC)

S3 Table Association of caffeine intake and caffeinated beverages with erectile dysfunction^β^ among men with and without hypertension NHANES 2001–2004. ^β^Erectile dysfunction was defined as “sometimes” or “never” able to maintain an erection for satisfactory sexual intercourse. ^‡^Adjusted for age, vigorous and moderate physical activity, smoking status, education, race/ethnicity, obesity (BMI ≥ 30 kg/m^2^), total water intake (plain and tap), total energy (continuous), alcohol (continuous). ^£^Approximately 170–375 mg/day of caffeine intake is equivalent to 2–3 cups of coffee. ^a^ *P* ≤ 0.05 ^b^ *P ≤* 0.01
(DOC)

S4 Table Association of caffeine intake and caffeinated beverages with erectile dysfunction^β^ among men with and without diabetes in NHANES 2001–2004. ^β^Erectile dysfunction was defined as “sometimes” or “never” able to maintain an erection for satisfactory sexual intercourse. ^‡^ Adjusted for age, vigorous and moderate physical activity, smoking status, education, race/ethnicity, obesity (BMI ≥ 30 kg/m^2^), total water intake (plain and tap), total energy (continuous), alcohol (continuous). ^£^Approximately 170–375 mg/day of caffeine intake is equivalent to 2–3 cups of coffee. ^a^ *P* ≤ 0.05 ^b^ *P ≤* 0.01
(DOC)
