# Supplementary material for: Role of Caffeine Intake on Erectile Dysfunction in US Men: Results from NHANES 2001-2004
Source: PLoS One. 2015 Apr 28;10(4):e0123547. doi: 10.1371/journal.pone.0123547 (PMC4412629; doi:10.1371/journal.pone.0123547)
Supplement: S1 Table — βErectile dysfunction was defined as “sometimes” or “never” able to maintain an erection for satisfactory sexual intercourse. †Model 1- Adjusted for age only. ‡Model 2- Adjusted for age, vigorous and moderate physical activity, smoking status, education, race/ethnicity, obesity (BMI ≥ 30 kg/m2), total water intake (plain and tap), total energy (continuous), alcohol (continuous). £ Approximately 170–375 mg/day of caffeine intake is equivalent to 2–3 cups of coffee. a P ≤ 0.05 b P ≤ 0.01 (DOC) [file pone.0123547.s001.doc]

**S1 Table**. Association of caffeine intake and caffeinated beverages with erectile dysfunction^β^ in NHANES 2001-2004 (n=3724).

| Variable | -ED/+ED | Model 1^†^  OR (95% CI) | Model 2^‡^  OR (95% CI) |
| --- | --- | --- | --- |
| Total caffeine intake (mg/day)^£^  1 Quintile (0-7)  2 Quintile (8-84)  3 Quintile (85-170)  4 Quintile (171-303)  5 Quintile (304-700)  *P_trend_* | 515/234  538/210  540/198  553/195  542/199 | 1.0  0.71 (0.50, 0.99)^a^  0.63 (0.43, 0.94)^a^  0.60 (0.40, 0.92)^a^  0.69 (0.49, 0.99)^a^  0.08 | 1.0  0.67 (0.46, 0.99)^a^  0.58 (0.37, 0.89)^a^  0.61 (0.38, 0.97)^a^  0.69 (0.45, 1.07)  0.19 |
| Coffee  No  Yes | 1,338/325  1,350/711 | 1.0  0.88 (0.70, 1.10) | 1.0  0.85 (0.67, 1.07) |
| Tea  No  Yes | 2,158/798  530/238 | 1.0  0.93 (0.69, 1.24) | 1.0  0.99 (0.67, 1.36) |
| Total soda  No  Yes | 994/550  1,694/486 | 1.0  1.1 (0.92, 1.32) | 1.0  1.05 (0.85, 1.30) |
| Energy and sport drinks  No  Yes | 2,588/1,020  100/16 | 1.0  0.49 (0.24, 1.00) | 1.0  0.43 (0.24, 0.77)^b^ |
| Coffee *plus* tea  No  Yes | 1,077/238  1,611/798 | 1.0  0.84 (0.67, 1.04) | 1.0  0.81 (0.65, 1.01) |
| Coffee *plus* tea and soda  No  Yes | 305/99  2,383/937 | 1.0  0.79 (0.58, 1.08 ) | 1.0  0.73 (0.52, 1.04) |
| Coffee *plus* tea, soda, and energy and sport drinks  No  Yes | 286/95  2,402/941 | 1.0  0.81 (0.58, 1.14) | 1.0  0.76 (0.52, 1.09) |

^β^Erectile dysfunction was defined as “sometimes” or “never” able to maintain an erection for satisfactory sexual intercourse.

^†^Model 1- Adjusted for age only.

^‡^Model 2- Adjusted for age, vigorous and moderate physical activity, smoking status, education, race/ethnicity, obesity (BMI ≥ 30 kg/m^2^), total water intake (plain and tap), total energy (continuous), alcohol (continuous).

^£^ Approximately 170-375 mg/day of caffeine intake is equivalent to 2-3 cups of coffee.

^a^*P* ≤ 0.05

^b^*P ≤* 0.01
