# Supplementary material for: Role of Caffeine Intake on Erectile Dysfunction in US Men: Results from NHANES 2001-2004
Source: PLoS One. 2015 Apr 28;10(4):e0123547. doi: 10.1371/journal.pone.0123547 (PMC4412629; doi:10.1371/journal.pone.0123547)
Supplement: S2 Table — βErectile dysfunction was defined as “sometimes” or “never” able to maintain an erection for satisfactory sexual intercourse. ‡Adjusted for age, vigorous and moderate physical activity, smoking status, education, race/ethnicity, total water intake (plain and tap), total energy (continuous), alcohol (continuous). £Approximately 170–375 mg/day of caffeine intake is equivalent to 2–3 cups of coffee. a P ≤ 0.05 b P ≤ 0.01 (DOC) [file pone.0123547.s002.doc]

**S2 Table**. Association of caffeine intake and caffeinated beverages with erectile dysfunction^β^ among normal weight and overweight/obese men in NHANES 2001-2004

| Variable | Normal Weight  OR (95% CI) | | Overweight/obesity  OR (95% CI) | |
| --- | --- | --- | --- | --- |
|  | -ED/+ED | Multivariable Model^‡^ | -ED/+ED | Multivariable Model^‡^ |
| Total caffeine intake (mg/day)^£^  1 Quintile (0-7g)  2 Quintile (8-84)  3 Quintile (85-170)  4 Quintile (171-303)  5 Quintile (304-700)  *P_trend_* | 181/61  148/55  157/47  165/42  133/51 | 1.0  1.27 (0.66, 2.42)  0.61 (0.29, 1.28)  0.83 (0.38, 1.84)  1.31 (0.69, 2.48)  0.84 | 325/172  385/154  380/149  385/151  404/147 | 1.0  0.51 (0.33, 0.80)^b^  0.53 (0.32, 0.85)^b^  0.51 (0.33, 0.79)^b^  0.54 (0.33, 0.91)^a^  0.08 |
| *P_interaction_* | 0.09 | | | |
| Coffee  No  Yes | 409/81  375/175 | 1.0  1.00 (0.60, 1.70) | 913/241  966/532 | 1.0  0.80 (0.61, 1.03) |
| *P_interaction_* | 0.64 | | | |
| Tea  No  Yes | 641/200  143/56 | 1.0  1.24 (0.71, 2.18) | 1,495/594  384/179 | 1.0  0.90 (0.61, 1.30) |
| *P_interaction_* | 0.44 | | | |
| Total soda  No  Yes | 298/152  486/104 | 1.0  0.65 (0.41,1.04) | 685/396  1,194/377 | 1.0  1.18 (0.92,1.53) |
| *P_interaction_* | 0.22 | | | |
| Energy and sport drinks  No  Yes | 748/249  36/7 | 1.0  1.08 (0.35, 3.36) | 1,817/765  62/8 | 1.0  0.17 (0.06, 0.47)^b^ |
| *P_interaction_* | 0.03 | | | |
| Coffee *plus* tea  No  Yes | 336/58  448/198 | 1.0  1.00 (0.58, 1.72) | 728/178  1,151/595 | 1.0  0.74 (0.58, 0.96)^a^ |
| *P_interaction_* | 0.53 | | | |
| Coffee *plus* tea and soda  No  Yes | 110/29  674/227 | 1.0  0.79 (0.41, 1.56) | 190/69  1,689/704 | 1.0  0.68 (0.43, 1.06) |
| *P_interaction_* | 0.45 | | | |
| Coffee *plus* tea, soda, and  energy and sport drinks  No  Yes | 102/26  682/230 | 1.0  0.89 (0.43, 1.83) | 179/68  1,700/705 | 1.0  0.68 (0.43, 1.07) |
| *P_interaction_* | 0.33 | | | |

^β^Erectile dysfunction was defined as “sometimes” or “never” able to maintain an erection for satisfactory sexual intercourse.

^‡^Adjusted for age, vigorous and moderate physical activity, smoking status, education, race/ethnicity, total water intake (plain and tap), total energy (continuous), alcohol (continuous).

^£^Approximately 170-375 mg/day of caffeine intake is equivalent to 2-3 cups of coffee.

^a^*P* ≤ 0.05

^b^*P ≤* 0.01
