# Supplementary material for: Role of Caffeine Intake on Erectile Dysfunction in US Men: Results from NHANES 2001-2004
Source: PLoS One. 2015 Apr 28;10(4):e0123547. doi: 10.1371/journal.pone.0123547 (PMC4412629; doi:10.1371/journal.pone.0123547)
Supplement: S3 Table — βErectile dysfunction was defined as “sometimes” or “never” able to maintain an erection for satisfactory sexual intercourse. ‡Adjusted for age, vigorous and moderate physical activity, smoking status, education, race/ethnicity, obesity (BMI ≥ 30 kg/m2), total water intake (plain and tap), total energy (continuous), alcohol (continuous). £Approximately 170–375 mg/day of caffeine intake is equivalent to 2–3 cups of coffee. a P ≤ 0.05 b P ≤ 0.01 (DOC) [file pone.0123547.s003.doc]

**S3 Table**. Association of caffeine intake and caffeinated beverages with erectile dysfunction^β^ among men with and without hypertension NHANES 2001 – 2004.

| Variable | No Hypertension  OR (95% CI) | | Hypertension  OR (95% CI) | |
| --- | --- | --- | --- | --- |
|  | -ED/+ED | Multivariable Model^‡^ | -ED/+ED | Multivariable Model^‡^ |
| Total caffeine intake (mg/day)^£^  1 Quintile (0-7g)  2 Quintile (8-84)  3 Quintile (85-170)  4 Quintile (171-303)  5 Quintile (304-700)  *P_trend_* | 302/52  305/65  302/51  316/57  293/66 | 1.0  1.04 (0.55, 1.98)  0.52 (0.26, 1.01)  0.66 (0.28, 1.54)  0.85 (0.41, 1.76)  0.51 | 212/182  232/145  238/145  237/138  249/133 | 1.0  0.47 (0.28, 0.79)^b^  0.54 (0.31, 0.96)^a^  0.56 (0.35, 0.89)^a^  0.58 (0.34, 0.99)^a^  0.13 |
| *P_interaction_* | 0.30 | | | |
| Coffee  No  Yes | 819/95  699/196 | 1.0  0.94 (0.57, 1.55) | 517/229  651/514 | 1.0  0.75 (0.55, 1.01)^a^ |
| *P_interaction_* | 0.85 | | | |
| Tea  No  Yes | 1,249/218  269/73 | 1.0  1.42 (0.80, 2.52) | 907/578  261/165 | 1.0  0.73 (0.53, 1.00)^a^ |
| *P_interaction_* | 0.03 | | | |
| Total soda  No  Yes | 526/132  992/159 | 1.0  1.14 (0.78, 1.67) | 467/418  701/325 | 1.0  0.99 (0.72, 1.35) |
| *P_interaction_* | 0.29 | | | |
| Energy and sport drinks  No  Yes | 1,145/284  73/7 | 1.0  0.43 (0.15, 1.18) | 1,141/734  27/9 | 1.0  0.59 (0.29, 1.26) |
| *P_interaction_* | 0.79 | | | |
| Coffee *plus* tea  No  Yes | 674/63  844/228 | 1.0  1.05 (0.61, 1.81) | 401/174  767/569 | 1.0  0.64 (0.47, 0.86)^b^ |
| *P_interaction_* | 0.46 | | | |
| Coffee *plus* tea and soda  No  Yes | 198/21  1,320/270 | 1.0  1.01 (0.53, 1.93) | 106/78  1,062/665 | 1.0  0.52 (0.31, 0.88)^a^ |
| *P_interaction_* | 0.20 | | | |
| Coffee *plus* tea, soda, and  energy and sport drinks  No  Yes | 181/19  1,337/272 | 1.0  1.08 (0.54, 2.16) | 104/76  1,064/667 | 1.0  0.53 (0.33, 0.91)^a^ |
| *P_interaction_* | 0.18 | | | |

^β^Erectile dysfunction was defined as “sometimes” or “never” able to maintain an erection for satisfactory sexual intercourse.

^‡^Adjusted for age, vigorous and moderate physical activity, smoking status, education, race/ethnicity, obesity (BMI ≥ 30 kg/m^2^), total water intake (plain and tap), total energy (continuous), alcohol (continuous).

^£^Approximately 170-375 mg/day of caffeine intake is equivalent to 2-3 cups of coffee.

^a^*P* ≤ 0.05

^b^*P ≤* 0.01
