# Supplementary material for: Role of Caffeine Intake on Erectile Dysfunction in US Men: Results from NHANES 2001-2004
Source: PLoS One. 2015 Apr 28;10(4):e0123547. doi: 10.1371/journal.pone.0123547 (PMC4412629; doi:10.1371/journal.pone.0123547)
Supplement: S4 Table — βErectile dysfunction was defined as “sometimes” or “never” able to maintain an erection for satisfactory sexual intercourse. ‡ Adjusted for age, vigorous and moderate physical activity, smoking status, education, race/ethnicity, obesity (BMI ≥ 30 kg/m2), total water intake (plain and tap), total energy (continuous), alcohol (continuous). £Approximately 170–375 mg/day of caffeine intake is equivalent to 2–3 cups of coffee. a P ≤ 0.05 b P ≤ 0.01 (DOC) [file pone.0123547.s004.doc]

**S4 Table**. Association of caffeine intake and caffeinated beverages with erectile dysfunction^β^ among men with and without diabetes in NHANES 2001 – 2004.

| Variable | No Diabetes  OR (95% CI) | | Diabetes  OR (95% CI) | |
| --- | --- | --- | --- | --- |
|  | -ED/+ED | Multivariable Model^‡^ | -ED/+ED | Multivariable Model^‡^ |
| Total caffeine intake (mg/day)^£^  1 Quintile (0-7g)  2 Quintile (8-84)  3 Quintile (85-170)  4 Quintile (171-303)  5 Quintile (304-700)  *P_trend_* | 482/171  501/152  502/150  514/140  505/146 | 1.0  0.63 (0.40, 0.99)^a^  0.53 (0.33, 0.84)^b^  0.51 (0.32, 0.82)^b^  0.58 (0.39, 0.87)^b^  0.01 | 33/63  37/58  38/48  39/55  37/53 | 1.0  0.72 (0.24, 2.14)  0.75 (0.24, 2.35)  0.95 (0.36, 2.51)  1.15 (0.42, 3.22)  0.57 |
| *P_interaction_* | 0.65 | | | |
| Coffee  No  Yes | 1,265/232  1,239/527 | 1.0  0.87 (0.66, 1.15) | 73/93  111/184 | 1.0  0.78 (0.43, 1.42) |
| *P_interaction_* | 0.43 | | | |
| Tea  No  Yes | 2,010/593  494/166 | 1.0  0.89 (0.59, 1.34) | 148/205  36/72 | 1.0  1.25 (0.63, 2.47) |
| *P_interaction_* | 0.37 | | | |
| Total soda  No  Yes | 919/426  1,585/333 | 1.0  0.89 (0.66, 1.19) | 75/124  109/153 | 1.0  1.47 (0.74, 2.94) |
| *P_interaction_* | 0.13 | | | |
| Energy and sport drinks  No  Yes | 2,405/745  99/14 | 1.0  0.47 (0.26, 0.84)^b^ | 183/275  1/2 | 1.0  0.73 (0.03, 13.5) |
| *P_interaction_* | 0.71 | | | |
| Coffee *plus* tea  No  Yes | 1,020/174  1,484/585 | 1.0  0.78 (0.60, 1.03) | 57/64  127/213 | 1.0  0.97 (0.50, 1.86) |
| *P_interaction_* | 0.96 | | | |
| Coffee *plus* tea and soda  No  Yes | 289/77  2,215/682 | 1.0  0.63 (0.43, 0.91)^a^ | 16/22  168/255 | 1.0  1.26 (0.53, 2.97) |
| *P_interaction_* | 0.24 | | | |
| Coffee *plus* tea, soda, and  energy and sport drinks  No  Yes | 270/73  2,234/686 | 1.0  0.65 (0.44, 0.96)^a^ | 16/22  168/255 | 1.0  1.26 (0.53, 2.97) |
| *P_interaction_* | 0.26 | | | |

^β^Erectile dysfunction was defined as “sometimes” or “never” able to maintain an erection for satisfactory sexual intercourse.

^‡^ Adjusted for age, vigorous and moderate physical activity, smoking status, education, race/ethnicity, obesity (BMI ≥ 30 kg/m^2^), total water intake (plain and tap), total energy (continuous), alcohol (continuous).

^£^Approximately 170-375 mg/day of caffeine intake is equivalent to 2-3 cups of coffee.

^a^*P* ≤ 0.05

^b^*P ≤* 0.01
